# CCN3/NOV serum levels in coronary artery disease (CAD) patients and its correlation with TNF-α and IL-6

**DOI:** 10.1186/s13104-023-06590-x

**Published:** 2023-11-02

**Authors:** Alaa Fadhil Jaafar, Reza Afrisham, Reza Fadaei, Vida Farrokhi, Nariman Moradi, Ali Abbasi, Nahid Einollahi

**Affiliations:** 1 Department of Clinical Laboratory Sciences, School of Allied Medical Sciences, Tehran University of Medical Sciences, Tehran, Iran; 2 Sleep Disorders Research Center, Kermanshah University of Medical Sciences, Kermanshah, Iran; 3 Department of Pharmacology, Vanderbilt University, Nashville, TN USA; 4 Department of Hematology, Faculty of Allied Medicine, Tehran University of Medical Sciences, Tehran, Iran; 5 Liver and Digestive Research Center, Research Institute for Health Development, Kurdistan University of Medical Sciences, Sanandaj, Iran; 6 Department of Cardiology, Dr Shariatee training and research Hospital, Tehran University of Medical Sciences, Tehran, Iran

**Keywords:** NOV/CCN3, Coronary artery disease, Inflammatory cytokines, Insulin resistance, Obesity

## Abstract

**Introduction:**

Dysregulation in the secretion of adipokines or adipocytokines plays a significant role in triggering a pro-inflammatory state, leading to endothelial dysfunction and insulin resistance, and ultimately elevating the risk of atherosclerosis and coronary artery disease (CAD). Previous studies have shown a link between NOV/CCN3 (an adipokine) and obesity, insulin resistance, and inflammation. However, no research has explored the relationship between CCN3 serum levels and CAD. Therefore, we conducted the first investigation to examine the correlation between CCN3 and CAD risk factors in patients.

**Methods:**

In a case-control study, we measured the serum levels of CCN3, IL-6, adiponectin, and TNF-α in 88 angiography-confirmed CAD patients and 88 control individuals using ELISA kits. Additionally, we used an auto analyzer and commercial kits to measure the biochemical parameters.

**Results:**

In patients with CAD, the serum levels of CCN3, TNF-α, and IL-6 were significantly higher compared to the control group, whereas lower levels of adiponectin were observed in the CAD group (P < 0.0001). A positive correlation was found between CCN3 and IL-6 and TNF-α in the CAD group ([r = 0.38, P < 0.0001], [r = 0.39, P < 0.0001], respectively). A binary logistic regression analysis showed the risk of CAD in the model adjusted (OR [95% CI] = 1.29 [1.19 − 1.41]), (P < 0.0001). We determined a cut-off value of CCN3 (3169.6 pg/mL) to distinguish CAD patients from the control group, with good sensitivity and specificity obtained for this finding (83.8% and 87.5%, respectively).

**Conclusion:**

This study provides evidence of a positive association between CCN3 serum levels and CAD, as well as inflammation markers such as IL-6 and TNF-α. These findings suggest that CCN3 may serve as a potential biomarker for CAD, and further investigations are necessary to validate this association and explore its potential use in clinical settings.

## Introduction

According to the World Health Organization’s report, the mortality rate attributable to cardiovascular diseases (CVD) is currently estimated to exceed 17 million individuals annually, with a projected increase to 23 million by 2023 [1, 2]. It is widely acknowledged that obesity and the associated metabolic alterations in adipose tissue are significantly linked to the development of cardiovascular diseases [3].

Adipose tissue is an important contributor to the onset of dyslipidemia, as well as endothelial and myocardial dysfunction. Dysregulation in the secretion of cytokines from adipose tissue, referred to as adipokines or adipocytokines, triggers a pro-inflammatory state, culminating in atherosclerosis, endothelial dysfunction, and insulin resistance, all of which heighten the susceptibility to coronary artery disease (CAD) [4–6]. The formation of sclerotic lesions has been shown to be linked to increased infiltration of inflammatory cells [7]. Such dysregulation in adipocytokine secretion is commonly observed in obesity, resulting from adipocyte hypertrophy and hyperplasia [3, 8]. Notable adipokines secreted by adipose tissue include tumor necrosis factor (TNF-α), adiponectin, leptin, interleukin-6 (IL-6), and resistin [9]. TNF-α, primarily secreted by adipose tissue, exhibits elevated gene and protein expression in the presence of obesity [10, 11], whereas only 30% of IL-6 production originates from adipose tissue [10]. The correlation between TNF-α, IL-6, and their involvement in obesity and metabolic disorders is well-established [12].

Conversely, NOV (nephroblastoma overexpressed protein)/CCN3 represents a distinct member of the CCN (cellular communication network factor) family and is a novel adipokine expressed in diverse tissues such as muscle, brain, and adrenal gland [13] and mostly in adipocytes and macrophages [14]. Recent discoveries have illuminated the pivotal involvement of NOV in facilitating the migration, adhesion, differentiation, and proliferation of diverse cell types [15–19], and it also involved in modulating inflammatory molecules [20, 21]. The adipokine NOV has been shown to heighten oxidative stress by suppressing antioxidants, impeding heme oxygenase (HO-1) function, and elevating free radical concentrations, resulting in a decline in vascular function [22]. In a separate investigation, a robust correlation has been established between plasma levels of NOV and adiposity metrics such as fat mass and body mass index (BMI) [14]. The expression of NOV within adipose tissue and its corresponding plasma levels demonstrate a marked increase in mice that have been subjected to a high-fat diet (HFD), in contrast to those that were fed a standard diet [14]. Mice fed a HFD that had the CCN3 gene knocked out exhibited improved glucose tolerance and insulin sensitivity. Furthermore, the absence of the NOV gene resulted in a noteworthy reduction in the expression of pro-inflammatory chemokines and cytokines [23]. In aggregate, these findings demonstrate a clear correlation between NOV and obesity, insulin resistance, and inflammation. In a recent study, Waldman et al. explored the impact of NOV on cardiometabolic function, revealing that inhibiting the expression of the NOV gene resulted in the reinstatement of anti-inflammatory genes, enhanced glucose tolerance, improved mitochondrial function, and heightened cardiac bioenergetics. Consequently, oxygen consumption increased, and heart metabolism was improved [22].

Given the established link between NOV/CCN3 serum levels and obesity-related disorders, insulin resistance, and inflammation, all of which are recognized as key risk factors for the development of coronary artery disease (CAD), it is plausible that NOV may serve as a promising biomarker for both the prognosis and management of CAD. To the best of our knowledge, prior research has not examined the association between NOV serum levels and CAD. Therefore, given its critical relevance in metabolic disorders, we conducted an unprecedented investigation into the serum levels of NOV among CAD patients, as well as its correlation with TNF-α, IL-6, and various biochemical parameters.

## Methods

### Study population

**Fig. 1:**
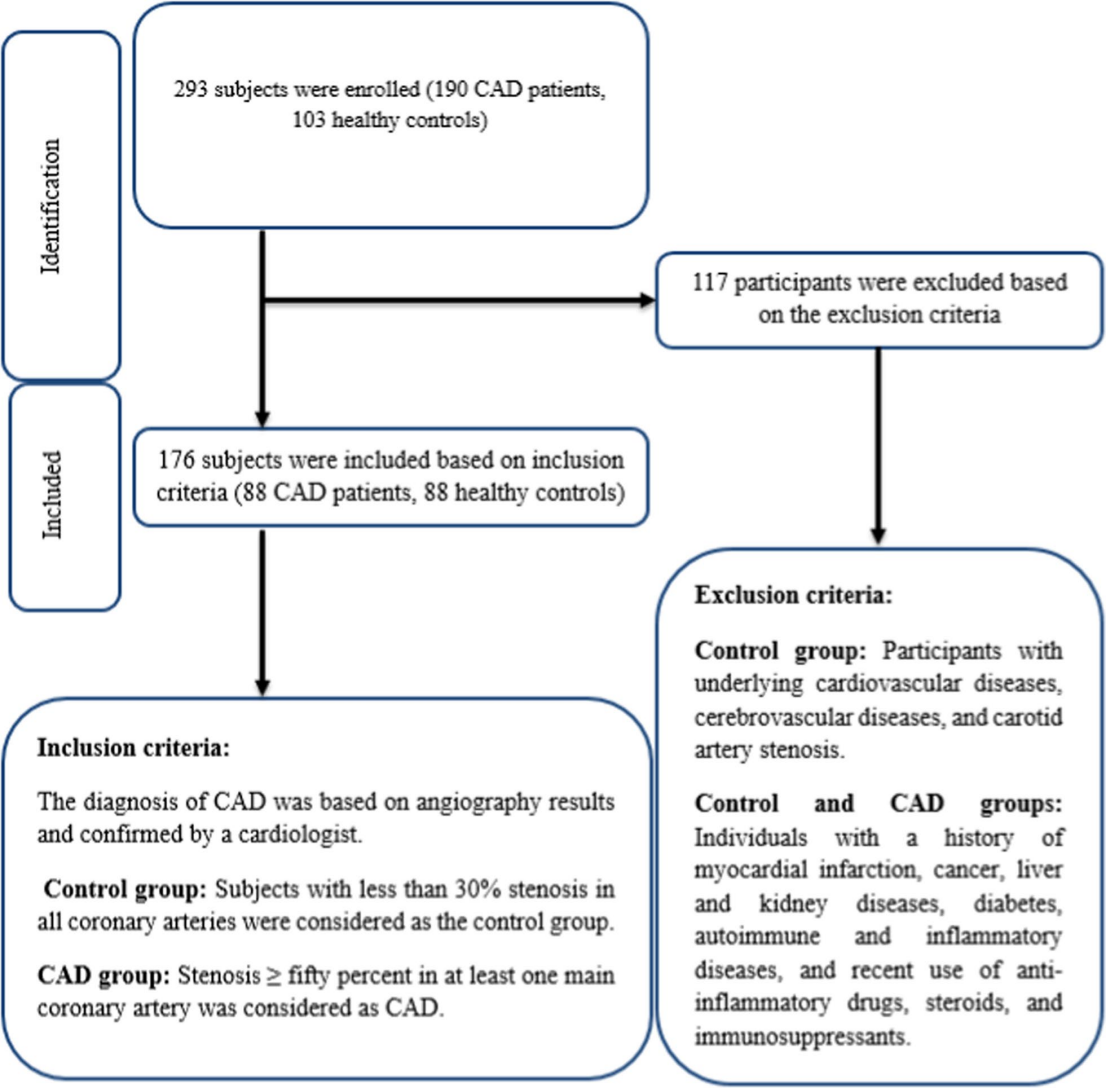
Flowchart of selection of subjects based on inclusion and exclusion criteria

In the current case-control investigation, a total of 176 participants aged between 45 and 75 years were selected, comprising 88 individuals in the control group and 88 subjects in the CAD group. The diagnosis of CAD was based on angiography results and confirmed by a cardiologist at Shariati Hospital in Tehran, Iran. Participants with underlying cardiovascular diseases, cerebrovascular diseases, and carotid artery stenosis were excluded from the control group. Subjects with less than 30% stenosis in all coronary arteries were considered as the control group. While, stenosis ≥ 50% in at least one main coronary artery was considered as CAD group. In addition, individuals with a history of myocardial infarction, cancer, liver and kidney diseases, diabetes, autoimmune and inflammatory diseases, and recent use of anti-inflammatory drugs, steroids, and immunosuppressants were also excluded. Flowchart of selection of subjects based on inclusion and exclusion criteria are shown in Fig. 1. Participants’ medical records were documented via a questionnaire. The protocols were performed in compliance with the Declaration of Helsinki and approved by the Ethics Committee of Tehran University of Medical Sciences (ID number: IR.TUMS.MEDICINE.REC.1402.066). In addition, informed consent was taken from all subjects.

### Biochemical and anthropometric measurements

The participants’ BMI was calculated using the standard formula of weight in kilograms divided by height in meters squared. Diastolic and Systolic blood pressure were taken while participants were seated using a standard sphygmomanometer. After an overnight fast, 10 milliliters of venous blood samples were obtained and biochemical parameters, including lipid profile (low-density and high-density lipoprotein-cholesterol [LDL-C, HDL-C], triglyceride [TG], total cholesterol [TC]), fasting blood glucose (FBG), creatinine (Cr), aspartate aminotransferase (AST), and alanine aminotransferase (ALT), were determined using an auto analyzer and commercial kits from Pars Azmoun, Iran. Fasting insulin levels were determined using an enzyme-linked immunosorbent assay (ELISA) kit from Monobind, USA, and the homeostasis model of insulin resistance (HOMA-IR) was calculated using the standard formula of fasting blood glucose in milligrams per deciliter multiplied by fasting blood insulin in micro international units per milliliter divided by 405.

### Cytokine and adipokine measurement

The quantification of serum levels of CCN3, adiponectin, IL-6, and TNF-α was performed using ELISA kits. The CCN3 kit (Aviscera Bioscience; USA) exhibited inter-assay and intra-assay coefficients of variation (CV) of 8–10% and 4–6%, respectively, and a detection limit of 31 pg/mL. Adiponectin levels were determined using a kit (Adipogen; South Korea) with inter- and intra-assay CV of 4.3% and 3.4%, respectively, and a lowest detectable range of 0.1 ng/mL. The evaluation kit for TNF-α and IL-6 serum levels (R & D Systems) had a lowest detectable range of 5.5 pg/mL and 0.11 pg/mL, respectively, with inter- and intra-assay CV values of 7.4 and 5.2 for TNF-α and 9.6 and 6.9 for IL-6.

### Statistical analysis

The statistical analysis was conducted using the SPSS21 software (Chicago, IL, USA), with a P-value less than 0.05 considered as statistically significant. Frequency and percentage were reported for categorical variables, and chi-square test was performed for their analysis. Continuous variables were tested for normality with the Kolmogorov-Smirnov test, and data with non-normal distribution were analyzed using the Mann-Whitney U test while the Student t-test was used for normal distribution data. Variables with normal and non-normal distribution were presented as mean ± standard deviation (SD) and median ± interquartile range (IQR), respectively. Moreover, analysis of covariance test (ANCOVA) was used to eliminate the impact of intervening variables on CCN3 levels. The correlation between CCN3 and biochemical as well as anthropometric parameters was evaluated through Pearson correlation analysis, and binary logistic regression was applied to evaluate its relationship with CAD risk. Furthermore, the cut-off value of CCN3 to distinguish CAD patients from the control group was tested using the receiver operating characteristic (ROC) curve.

## Results

### Anthropometric and biochemical parameters

The study results, presented in Table 1, indicate that age (P = 0.621) and BMI (P = 0.361) did not differ significantly between the healthy and CAD groups. However, diastolic (P = 0.020) and systolic (P = 0.009) blood pressure were found to be significantly higher in the CAD group. Furthermore, the CAD group exhibited significantly higher levels of insulin (P = 0.001), HOMA-IR (P = 0.001), and AST (P = 0.004) compared to the control group, while no significant differences in FBG (P = 0.727), ALT (P = 0.094), and creatinine (P = 0.200) values were observed. Lipid profile markers, including TG (P = 0.001), TC (P = 0.0001), and LDL-C (P = 0.007), were also elevated in CAD patients, with the exception of HDL-C (P = 0.860).

**Table 1:** Basic anthropometric, immunological and biochemical characteristics of 88 control people and 88 CAD patients

Variables	Non-CAD	CAD	*P*-value
Age (year)	57.51 ± 8.11	58.15 ± 8.13	0.62
BMI (kg/m2)	26.45 ± 3.72	25.92 ± 3.60	0.36
SBP (mmHg)	128.3, 117.94–140	130.5, 125-142.75	0.008
DBP (mmHg)	79, 70-85.5	80, 78–90	0.008
FBG (mg/dL)	94.21 ± 12	94.85 ± 11.11	0.72
Insulin (µU/mL)	3.6, 2.12–6.47	5.75, 3.4–8.97	0.001
HOMA-IR	0.8, 0.53–1.46	1.37, 0.76–2.21	0.001
TG (mg/dL)	121.16 ± 47.91	146.05 ± 48.20	0.001
TC (mg/dL)	161.98 ± 40.06	186.71 ± 44.30	< 0.001
LDL-C (mg/dL)	98.51 ± 32.50	112.14 ± 30.48	0.007
HDL-C (mg/dL)	44.30 ± 8.03	44.06 ± 8.92	0.86
Creatinine (mg/dL)	1.13, 0.18	1.17 ± 0.16	0.20
AST (U/L)	18, 14.82–23.33	21, 16.25-27	0.007
ALT (U/L)	20, 14.05–26.22	24, 15.05-28	0.096
CCN3 (pg/mL)	2869.69, 2723.44-3060.43	4211.19, 3327.62-4763.15	< 0.001
TNF-alpha (pg/mL)	22.94 ± 7.37	26.92 ± 7.05	0.001
IL-6 (pg/mL)	6.15, 4.52–7.4	8.1, 5.8–11.5	< 0.001
Adiponectin (µg/mL)	10.6, 7.57–13.2	8.6, 6.4–10.9	0.004

Data are presented as mean ± SD and median (IQR)

*CCN3* cellular communication network factor 3, *BMI* Body mass index, *SBP* systolic blood pressure, *DBP* diastolic blood pressure, *FBS* fasting blood sugar, *HOMA-IR* Homeostatic model assessment for insulin resistance, *AST* Aspartate aminotransferase, *ALT* Alanine aminotransferase, *TG* Triglyceride, *TC* total cholesterol,* LDL-C* Low density lipoprotein-cholesterol, *HDL-C* High density lipoprotein-cholesterol, *IL-6* Interleukin 6, *TNF-alpha* Tumor necrosis factor-alpha

### Cytokine and adipokines levels

**Fig. 2:**
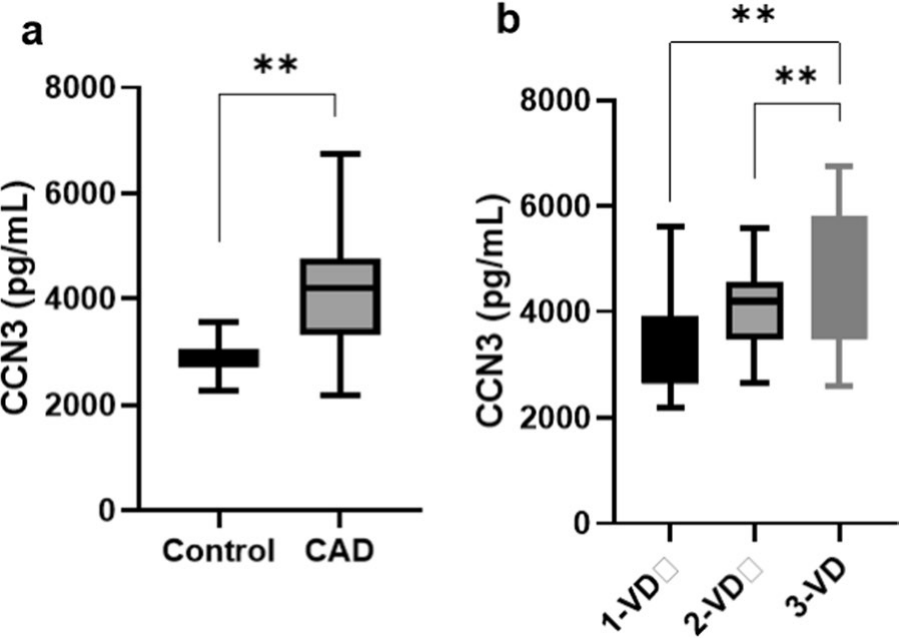
Serum levels of CCN3 (**a**) were higher in the CAD group than the control group (**b**) and in the subgroups in terms of vessel disease, were higher in the 3-vessel disease compared to the 2-vessel disease and 1-vessel disease. Data are presented as median (IQR).

In individuals with CAD, the mean serum level of CCN3 was significantly elevated at 4229.17 ± 1131.42 pg/mL, compared to the healthy people with a mean of 2909.60 ± 400.03 pg/mL (P = 0.0001) (Fig. 2a). Further subgroup analysis revealed that those with three-vessel stenosis had a higher CCN3 serum level than those with one-vessel (P = 0.0001) and two-vessel (P = 0.0001) stenosis (Fig. 2b). Interestingly, no significant sex-based differences were observed in CCN3 levels between the CAD (22 female, 58 male) and control (16 female, 64 male) groups (P = 0.26).

Additionally, the CAD group had higher levels of IL-6 and TNF-α with means of 8.68 ± 3.66 and 26.92 ± 7.05 pg/mL, respectively, compared to the control group (6.20 ± 2.39 and 22.94 ± 7.37 pg/mL, respectively) with almost a significant P-value of 0.001. Moreover, a lower level of adiponectin was observed in the CAD group (8.815 ± 2.9 µg/mL) compared to the control group (10.955 ± 4.93 µg/mL) with a significant P-value of 0.001.

### Correlation of CCN3 serum levels with the risk factors of CAD

The results of the Pearson correlation analysis, as demonstrated in Tables 2 and 3, revealed a significant positive correlation between CCN3 and IL-6 as well as TNF-α levels in the CAD group ([r = 0.38, P < 0.0001], [r = 0.39, P < 0.0001]), respectively. Conversely, the correlation between CCN3 and both IL-6 and TNF-α levels was not noteworthy in the control subjects. Furthermore, no significant correlation was observed between CCN3 and the other variables presented.

**Table 2:** The correlation analysis of various variables with CCN3 in the control group

Variables	r	*P*-value
Age	0.07	0.52
BMI	0.16	0.15
SBP	0.02	0.80
DBP	0.06	0.56
FBG	0.04	0.71
Insulin	0.03	0.76
HOMA-IR	0.04	0.69
TG	0.04	0.68
TC	0.15	0.17
LDL-C	0.12	0.26
HDL-C	− 0.08	0.44
Creatinine	0.01	0.89
AST	0.12	0.26
ALT	0.01	0.88
TNF-alpha	0.05	0.61
IL-6	0.08	0.48
Adiponectin	− 0.12	0.27

**Table 3:** The correlation analysis of various variables with CCN3 in the CAD group

Variables	r	*P*-value
Age	− 0.17	0.11
BMI	0.02	0.82
SBP	− 0.02	0.83
DBP	− 0.04	0.72
FBG	0.17	0.11
Insulin	0.01	0.87
HOMA-IR	0.04	0.71
TG	0.06	0.56
TC	− 0.06	0.56
LDL-C	0.03	0.76
HDL-C	− 0.08	0.46
Creatinine	− 0.02	0.85
AST	0.16	0.13
ALT	0.02	0.81
**TNF-alpha**	**0.39** ^**^	**< 0.001**
**IL-6**	**0.38 ** ^**^	**< 0.001**
Adiponectin	− 0.15	0.17

* *P* < 0.05; ** *P* < 0.001

### Correlation of CCN3 serum level with CAD

A binary logistic regression analysis was performed to examine the probability of CAD occurrence with every 100-unit variation in CCN3 serum levels, which yielded consistent and noteworthy results in both the unadjusted (OR [95% CI] = 1.29 [1.188 − 1.41]) and adjusted models accounting for age, gender, and BMI (OR [95% CI] = 1.29 [1.190 − 1.41]) (P < 0.0001) (refer to Table 4).

**Table 4:** Binary logistic regression for odd ratio of CAD status according to 100-unit change in CCN3.

Model	B	S.E.	Wald	df	P	Odd ratio (B)	95% CI.for odd ratio (B)	
							Lower	Upper
Crude model	0.25	0.04	34.65	1	< 0.001	1.29	1.18	1.41
Model-1	0.26	0.04	34.97	1	< 0.001	1.29	1.19	1.41
Model-2	0.24	0.04	27.03	1	< 0.001	1.27	1.16	1.39

Model-1: adjusted for age, sex and BMI

Model-2: adjusted for SBP, DBP, TG, TC, LDL-C, and HDL-C

**Fig. 3:**
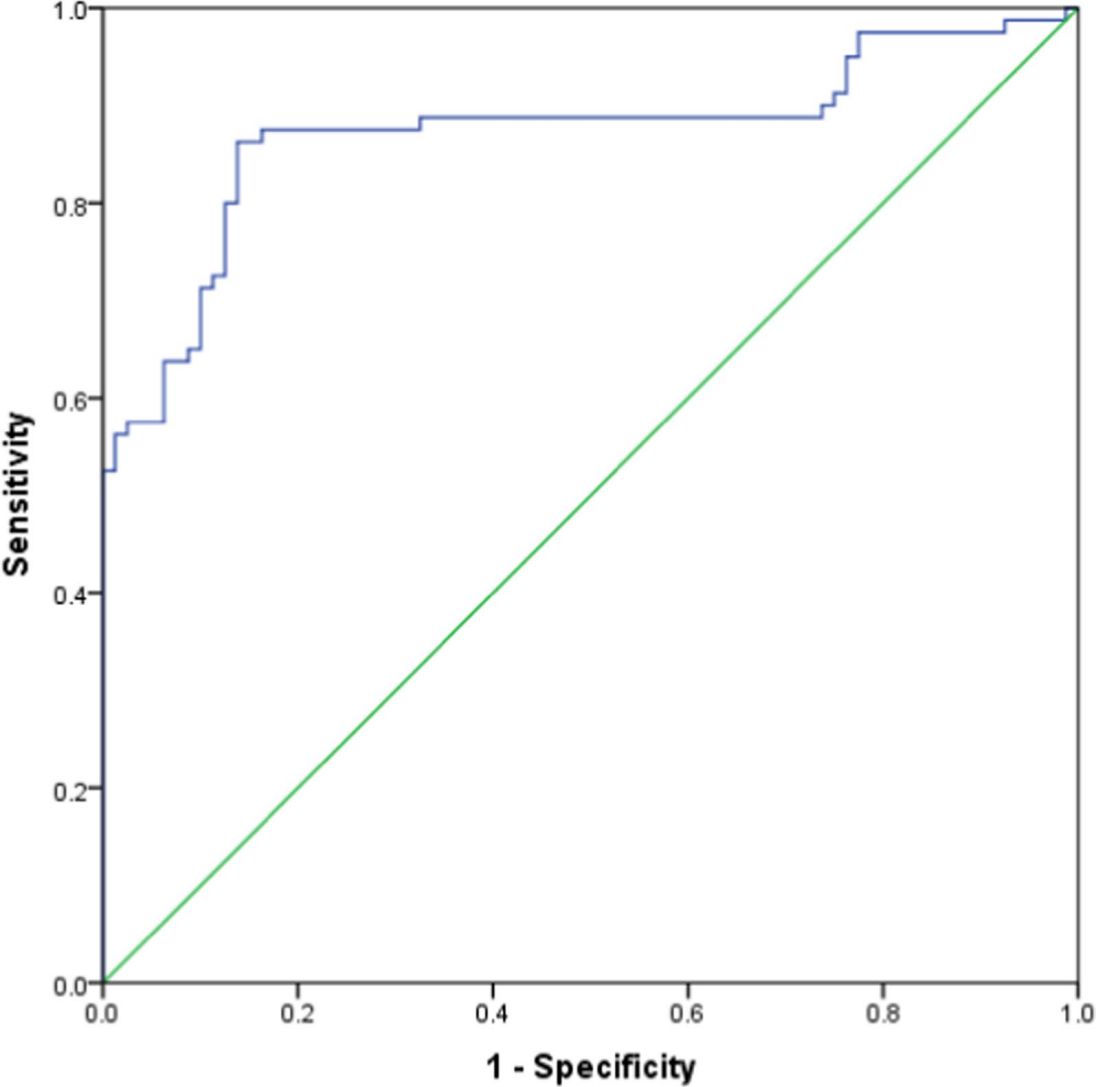
ROC curve for diagnosis of CAD according to CCN3 serum levels. a cut-off value of CCN3 (3169.6 pg/mL) was identified to distinguish between CAD patients and the control group, demonstrating good sensitivity and specificity (83.8% and 87.5%, respectively). The area under the curve was calculated as 0.87 [0.81–0.93 (95% Cl), P < 0.0001]

Based on the ROC curve analysis, a cut-off value of CCN3 (3169.6 pg/mL) was identified to distinguish between CAD patients and the control group, demonstrating good sensitivity and specificity (83.8% and 87.5%, respectively). The area under the curve was calculated as 0.87 [0.81–0.93 (95% Cl)] and exhibited a significant p value of less than 0.0001 (refer to Fig. 3).

## Discussion

In this current investigation, we have established the link between serum levels of CCN3 and CAD, marking the first of its kind to explore such a relationship. Prior research has delved into the association of various members of the CCN family (ranging from CCN1 to CCN6) with metabolic disorders, encompassing conditions such as obesity, diabetes, insulin resistance, and non-alcoholic fatty liver [24–26]. To the best of our knowledge, prior research has established the involvement of CCN1 and CCN4 in the pathogenesis of atherosclerosis [27, 28]. Moreover, previous studies have linked CCN3 to various disorders such as insulin resistance, obesity, non-alcoholic fatty liver disease, and inflammation [29, 30], however, the relationship between CCN3 serum levels and CAD has not been explored thus far. Given the significance of CCN family members as novel adipokines involved in metabolic processes, our study aimed to investigate the potential of CCN3 as a novel biomarker for prognosis and disease management in CAD patients.

The results of our study demonstrate a significant upregulation of serum CCN3 in CAD patients compared to controls, with a positive correlation between CCN3 levels and CAD risk. Notably, a CCN3 cutoff value was established with good sensitivity and specificity (> 80%). Additionally, we observed a positive correlation between CCN3 and IL-6 and TNF-α in CAD patients, consistent with previous studies highlighting the regulatory role of CCN3 in metabolic processes. For instance, Martinerie et al. [23] demonstrated improved glucose tolerance and insulin sensitivity, as well as prevention of adipose tissue accumulation and obesity, in CCN3 gene knockout mice fed with HFD. Notably, CCN3 knockout led to increased expression of PGC1-α in brown adipose tissue, which, in cooperation with PPAR-γ, activates the UCP-1 promoter to reduce fat mass and enhance energy consumption. Thus, our findings suggest that CCN3 may serve as a potential therapeutic target for improving insulin sensitivity and preventing obesity in CAD patients [23]. Furthermore, in recent investigations carried out in 2019 and 2022, the suppression of CCN3 expression has been shown to have a beneficial effect on activating the HO-1/PGC-1α pathway, leading to an improvement in cardiac function. The activation of PGC-1α is known to impede adipose tissue hypertrophy and enhance cardiovascular health by augmenting mitochondrial function, refining oxygen consumption, and modulating metabolism [22, 31]. In contrast, upon CCN3 inhibition, AKT is stimulated, thereby inducing mitophagy. This process holds promise in enhancing oxygen consumption and insulin sensitivity, thereby improving obesity and vascular function [22]. Furthermore, Martinerie et al. demonstrated that the lack of CCN3 gene expression can elicit a greater production of M2 macrophages in contrast to M1 macrophages, resulting in a reduction of chemokines and inflammatory cytokines [23]. The observed positive correlation in our study between elevated CCN3 serum levels and an increase in inflammatory cytokines demonstrates the potential of CCN3 to indirectly influence the expression and secretion of such cytokines from adipose tissue [23].

Pakradouni et al. [14] conducted a study investigating the correlation between CCN3 serum levels and obesity. The study demonstrated that CCN3 was upregulated in adipose tissue of obese humans and mice, and there was a positive association between obesity-related inflammation and CCN3 plasma levels. Consistent with our findings, no significant correlation was observed between CCN3 plasma levels and LDL-C, HDL-C, TC, and blood glucose in individuals with metabolic disorders. However, in contrast to our results, Pakradouni’s research identified a positive and significant correlation between CCN3 levels and BMI, as well as a noteworthy impact of gender on CCN3 plasma levels [14]. Despite the well-established association between high BMI and cardiovascular diseases, our study did not identify a significant relationship between the two. Possible reasons for this disparity include the inclusion of individuals with similar BMI values in both the CAD and control groups, as well as the relatively small sample size employed in this investigation.

Notably, our findings lend support to the notion that CCN3 levels in CAD patients increase independently of obesity and insulin resistance parameters, thus potentially serving as a robust biomarker for the prognosis of CAD patients. Nonetheless, to draw a more definitive conclusion, a larger cohort of subjects must be studied.

## Conclusion

Our findings reveal, for the first time, a notable elevation in serum CCN3 levels among CAD patients in comparison to the control group. Notably, our study indicates a significant positive association between CCN3 serum levels and the progression of CAD. Furthermore, the identification of a significant correlation between CCN3 and inflammatory cytokines (IL-6 and TNF-α) provides further insight into the potential role of CCN3 in the pathogenesis of CAD. Given the robust association between CCN3 and CAD, which persists even after controlling for variables such as age, gender, and BMI, it is plausible that CCN3 may serve as a promising prognostic marker for CAD.

## Data Availability

The datasets used and/or analysed during the study available from the corresponding author on reasonable request.
